# Exercise induced plasma volume expansion lowers cardiovascular strain during 15-km cycling time-trial in acute normobaric hypoxia

**DOI:** 10.1371/journal.pone.0297553

**Published:** 2024-02-02

**Authors:** Felipe Gorini Pereira, Andrew M. Greenfield, Matthew Kuennen, Trevor L. Gillum

**Affiliations:** 1 Department of Kinesiology, California Baptist University, Riverside, CA, United States of America; 2 Department of Kinesiology, Indiana University, Bloomington, IN, United States of America; 3 Department of Exercise Science, High Point University, High Point, NC, United States of America; Università degli Studi di Milano: Universita degli Studi di Milano, ITALY

## Abstract

The purpose of our study was to assess the influence of a single high-intensity interval exercise (HIIE) bout in normoxia on plasma volume (PV) and consequent cycling performance in normobaric hypoxia (0.15 F_i_O_2_, simulating ~2,500 m). Eight males (VO_2peak_: 48.8 ± 3.4 mL/kg/min, 24.0 ± 1.6 years) completed a hypoxic 15 km cycling time trial (TT), followed by a crossover intervention of either HIIE (8x4 min cycling bouts at 85% of VO_2peak_) or CON (matched kJ production from HIIE at 50% of VO_2peak_). 48 hours post intervention, an identical TT was performed. Cardiovascular parameters were measured via impedance cardiography during each TT. Changes in PV was measured 24 and 48 hours post HIIE and CON. HIIE increased PV at 24 (4.1 ± 3.9%, *P* = 0.031) and 48 (6.7 ± 1.7, *P* = 0.006) hours post, while no difference was observed following the CON (1.3 ± 1.1% and 0.3 ± 2.8%). The higher PV led to an increased stroke volume (*P* = 0.03) and cardiac output *(P* = 0.02) during the hypoxic TT, while heart rate was not changed (*P* = 0.49). We observed no changes in time to completion (−0.63 ± 0.57 min, *P* = 0.054) and power output (7.37 ± 7.98 W, *P* = 0.078) between TTs. In the absence of environmental stress, a single bout of HIIE was an effective strategy to increase PV and reduce the cardiovascular strain during a cycling TT at moderate simulated altitude but did not impact hypoxic exercise performance.

**Trial registration: Clinical Trials ID**: NCT05800808

## Introduction

Arterial oxygen saturation and content is reduced during dynamic exercise in hypoxic conditions, necessitating increased blood flow in order to meet the augmented energetic demand of the locomotor muscles [1, 2]. Moreover, respiratory muscle fatigue is exacerbated in this environment, as previous work suggests that the work of breathing during intense exercise is augmented during hypoxia exposure [3], congruently forcing respiratory muscles to perform increased work output with limited blood flow. These detrimental factors lead to an exuberant increase in cardiovascular demand, necessitating an increased heart rate (HR) to offset the lower oxygen saturation, in an attempt to maintain adequate cardiac output ($\dot{Q}$) in light of an accelerated rate of peripheral fatigue [4]. For this reason, unacclimated individuals experience decreased work capacity during acute altitude exposure [5–7]. Many turn to conventional altitude acclimatization strategies that incorporate intermittent altitude exposures. Best practice guidelines currently recommend 14–19 days for endurance performance [8, 9], but this most likely varies based upon the hours associated with a given level of hypoxia [10]. However, acclimatization is often impractical due to the amount of time required and a lack of access to proper training facilities. As a result, military personnel, wildland firefighters, collegiate, and recreational athletes often perform physical activities at moderate altitude in an unacclimated state.

Interest in novel strategies that could help to mitigate altitude-associated declines in exercise capacity has grown over the years. Elevations in plasma volume (PV) enhance end diastolic ventricular filling and thus $\dot{Q}$, contributing to reductions in cardiovascular strain at a given exercise intensity [11]. Indeed, acute PV expansion has been shown to reduce cardiovascular strain during sea level exercise [12, 13]. It is also thought to improve prolonged endurance exercise performance, which under normal conditions is characterized by significant reductions in PV [14]. Studies have utilized intravenous infusions of pharmacological agents (i.e. dextran, Gelofusine, and glycerol) to increase PV, which has been shown to improve maximal oxygen uptake ($\dot{V}O_{2\mathrm{peak}}$) and prolong exercise-time to exhaustion [15, 16]. Importantly, despite noticeable reductions in hemoglobin (Hb) and hematocrit (Hct) concentrations, these improvements in performance are well established. It has been suggested that perhaps the elevations in SV and $\dot{Q}$ that accompany expansion of PV may offset the unfavorable effects of a reduction in Hb concentration, thereby allowing muscle oxygen delivery to be preserved during high intensity exercise [12, 17]. Given these findings, an expansion of PV may be a beneficial strategy to partially mitigate the deleterious effects of hypoxia on the cardiovascular system.

Rather than focusing on PV expansion via pharmacological manipulation that may have limited application outside laboratory environments, we question if high intensity interval exercise (HIIE) may be an adequate stimulus to promote hypervolemia in a recreationally active cohort. Green et al. demonstrated a 12% increase in PV following three days of intermittent supramaximal exercise bouts [18]. Furthermore, Gillen et al. described a PV increase of ~10% 24 hours following a single session of eight, 4-min intervals at 85% of $\dot{V}O_{2\mathrm{peak}}$ [19]. These findings suggest that HIIE could be a practical and time-effective strategy to induce PV expansion, as compared to the utilization of pharmacological agents and/or heat-exposure strategies. However, to our knowledge, no reports have attempted to explore if HIIE could be utilized as a rapid means to expand PV and if these effects might confer benefits during subsequent exercise performance in moderate hypoxia. To this end, we have characterized the PV expansion response from a single HIIE protocol [19] and ascertained the impact of said hypervolemic response on time-trail (TT) performance in normobaric hypoxia (F_i_O_2_: 0.15, ~2,500 m). We hypothesized that a single HIIE session would increase PV and attenuate cardiovascular strain during exercise in hypoxia, as evidenced by reductions in HR and elevations in SV and $\dot{Q}$. Furthermore, we hypothesized that these changes would contribute to a reduced time-to-completion in a 15 km, self-paced cycling TT.

## Methods and materials

Eight recreationally active males were recruited to participate in this study (Table 1). Sample size was determined with G*Power 3.1.9.4 [20] using ΔPV (evidenced by variations in Hct & Hb) from a recreationally active pool of subjects 24 hours following a single running session of eight, 4-min intervals at 85% of $\dot{V}O_{2\mathrm{peak}}$ (ΔPV = 10.1 ± 0.9%) [19]. From those data, it was determined that an α-level of *P* ≤ 0.05, seven participants would provide an 80% probability (i.e., 1 - β) of detecting a statistically significant change in PV following the prescribed HIIE bout.

**Table 1:** Subject demographics (*N* = 8).

	Mean	SD
Age (years)	24.0	1.8
Weight (kg)	80.2	9.7
Height (cm)	182.3	7.1
BMI	23.6	3.5
Body Fat (%)	12.3	4.2
$\dot{V}O_{2\mathrm{peak}}$ (ml/kg/min^-1^)	48.8	3.4
Peak Power (W)	395	48.6

Data are represented as mean ± SD.

Participants were screened for environmental exposure to heat and hypoxia and were not frequent hot bath or sauna users in the three months prior to data collection. Experimental trials were performed in late fall through spring months (Riverside, CA) where weather was not favorable for natural heat acclimatization. Further, the elevation of Riverside, CA is 252 meters above sea level. Participants were screened for cardiovascular and metabolic disease using the American College of Sports Medicine risk stratification guidelines and a Physical Activity Readiness Questionnaire [21]. Participants were not taking any form of medication or supplements throughout the duration of the study. Study staff verbally informed each participant of research procedures, and each gave written informed consent as declared in the 1964 Declaration of Helsinki. All protocols were approved by the Institutional Review Board of California Baptist University (IRB 072-1819-EXP) and the study was registered on clinnicaltrials.gov (NCT05800808). All participants were assigned an identification number, which was then used when recording and analyzing data. A code list containing the participant’s name and identification numbers were stored separately in a locked filing cabinet and was only available to investigators.

**Fig 1:**
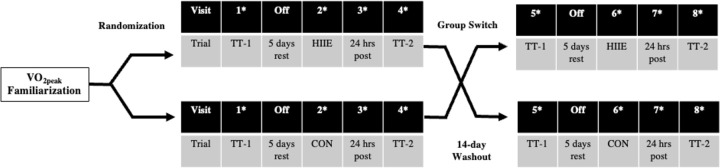
Study schematic and design. Study participants were randomized into HIIE or CON intervention groups. On visits 1 and 4, both groups completed hypoxic TT-1 and TT-2 for their respective intervention. Following washout and group switch, on visits 5 and 8, both groups completed hypoxic TT-1 and TT-2 for their remaining intervention. *Denotes visits where blood draws occurred.

A crossover design was utilized to assess the effects of exercise-induced PV expansion on cycling performance in hypoxia. All participants completed HIIE and a control intervention (CON) in a counterbalanced order. HIIE consisted of 8 x 4 min bouts of cycling exercise at 85% of $\dot{V}O_{2\mathrm{peak}}$ with 4 min rest between intervals (19). CON consisted of cycling at 50% $\dot{V}O_{2\mathrm{peak}}$ and the overall workload was matched to the HIIE intervention in kilojoules (kJ) produced, resulting in similar workout output for both training conditions (HIIE = 546 ± 84.1kJ; CON = 547 ± 82.3 kJ). The duration of the CON bout was extended to properly match HIIE kJ production (HIIE: 60 ± 0.0 min; CON: 81 ± 7.2 min). The HIIE and CON training bouts were performed in a thermoneutral and normoxic environment (20°C, 35% RH, F_i_O_2_ = 0.2093). Two-15 km, self-paced cycling TTs were performed before and after each training intervention (Fig 1). The first TT occurred 5 days before the training intervention (HIIE or CON) and the second TT occurred 48 hours post intervention. Interventions were separated by 14 days to ensure sufficient washout of any training effect (Fig 1) as data have suggested retention of expanded PV may last for 7–14 days [22]. During washout, participants were instructed to continue their normal exercise routine.

### Preliminary testing

During preliminary testing, body composition was assessed via a 3-site skinfold (chest, abdomen, thigh) measurement. Each site was measured twice in a rotational order and the mean values were summed and used to estimate body density and fat using a standardized regression equation [23]. Aerobic capacity was measured through indirect calorimetry (Parvo Medic, Sandy UT). Participants performed incremental exercise on a cycle ergometer (Monark LC7 TT, Monark, Sweden), where the exercise workload increased 20 Watts per minute following a starting workload of 50 Watts, and pedal cadence was maintained at or above 80 revolutions per minute. For the test to be considered maximum, three of the following $\dot{V}O_{2\mathrm{peak}}$ criteria were met: 1) $\dot{V}O_{2}$ plateau (less than 0.250 L/min), despite increase in workload, 2) rating of perceived exertion (RPE) ≥ 17, 3) respiratory exchange ratio > 1.10, and 4) HR ≥ 10 bpm of age-predicted max. Maximal workload was recorded and used to determine exercise workload for HIIE and CON training. After resting for ~15-minutes, participants performed a familiarization 15 km TT in room temperature normoxia (20 C, 35% RH, F_i_O_2_: 0.20). This was done to accustom participants to the equipment and reduce the likelihood of learning effects causing data bias.

### Hypoxic TTs

The first hypoxic TT within each condition served as a baseline measure of performance for each participant (Fig 1). Participants abstained from strenuous exercise and alcohol 24 hours prior to each TT. Participants were required to maintain a food log 36 hours prior to the first TT, and then were requested to replicate it for subsequent visits. Upon arrival to the laboratory, participants voided their bladder and provided a urine sample for hydration assessment. Urine specific gravity (USG) was analyzed using a handheld refractometer (ATAGO USA, Bellevue, WA) and adequate hydration was determined as a specified cutoff of USG <1.020 [24]. Following 20 min of seated rest, a 3 mL blood sample was drawn from the antecubital vein. Three microcuvette tubes were prepared and centrifuged (Bio Lion, Manassas, VA) for later hematological analysis of Hct. The remainder of the blood was immediately dispensed into an EDTA coated vacutainer and analyzed for Hb concentration via photospectrometry (UNICO, Princeton, NJ).

Nitrogen dilution was used to create a hypoxic environment inside an environmental chamber (Cascade Solutions, El Cajon, CA), which was monitored through two independent O_2_ sensors (BioSpherix P360) set to maintain F_i_O_2_ levels of 0.15 (simulating ~ 2,500 m of elevation).

Participants wore standard exercise attire which included cycling shorts and a t-shirt. Participants were then instrumented with an impedance cardiography apparatus (PhysioFlow, Nanatec Biomedical, France) to measure hemodynamic variables (HR, $\dot{Q}$, SV) The anatomical sites required for impedance measurement were cleansed with isopropyl alcohol and shaved (if needed) to ensure proper contact and conductance. Prior to exercise, resting blood pressure was recorded after 20 mins of seated rest. Resting blood pressure was recorded to ensure proper calibration for impedance cardiography measurement. Following instrumentation, participants entered the environmental chamber (20°C, 35% RH) and performed a 10 min warmup by cycling at a power output corresponding to 50% of their individual $\dot{V}O_{2\mathrm{peak}}$.

Upon completion of the warmup, participants performed a 15 km TT as quickly as possible. Throughout the TT, distance covered was made available to participants, but time, speed, cadence, and power were blinded. All participants were instructed to complete the TT as quickly as possible and received verbal encouragement. Blood lactate (Nova Biomedical, Waltham, MA) was measured before (BL_Rest_) and immediately after (BL_peak_) each TT from a finger capillary whole blood sample. RPE, which ranged from 6 (no exertion) to 20 (maximal exertion), was also measured via the Borg scale [25] and SpO_2_ was measured at the index finger using a standard pulse oximeter (Nellcor Bedside, Nellcor Inc. Hayward, CA).

### PV expansion analysis

Blood samples were collected following 20 min seated rest prior to exercise for each TT and testing condition (HIIE and CON). A total of 8 blood samples (4 per intervention) were collected throughout the study as depicted in Fig 1. Hb and Hct were measured to determine changes in PV, as described by Dill and Costill [26] using the following equation:

$$\Delta PV(\%)=\frac{{Hb}_{pre}X(1-{Hct}_{post})}{{Hb}_{post}X(1-{Hct}_{pre})}$$

Using the above equation, we calculated ΔPV (%) for three distinct time-points: Pre-training (i.e., prior to HIIE/CON), 24 hours post training, and 48 hours post training (i.e., just prior to TT-2).

### Statistical analysis

Data were analyzed using GraphPad Prism version 9.0.0 (Prism, San Diego, CA) statistical software. Normality of dependent variables were determined using a Shapiro–Wilk test and homogeneity of variance using Levene’s test. An alpha level of 0.05 was set a priori. Changes in PV were analyzed using a two-factor (intervention x time) repeated measures ANOVA. Averaged hemodynamic parameters (SV (mL/beat), HR (bpm), and $\dot{Q}$ (L/min)), as well as perceptual (RPE_final_) and physiological (SpO_2_ (%), BL_Rest_ (mmol/L), BL_peak_ (mmol/L), and USG) responses during the TT were analyzed using a two-factor (intervention x trial) repeated measure ANOVA. We performed the Holm-Sidak post-hoc procedure to determine where differences existed if the ANOVA revealed a significant interaction or main effect. Mean differences of TT performance variables (time to completion (min) and average power output (W)) were analyzed using two-tailed Wilcoxon matched-pairs, signed rank tests. All data values are reported as mean ± standard deviation (SD) or mean difference ± SD. Cohen’s d was calculated to determine effect size with a value of 0.2, 0.5, 0.8 representing small, medium, and large effects; respectively [27].

## Results

### Plasma volume expansion

**Fig 2:**
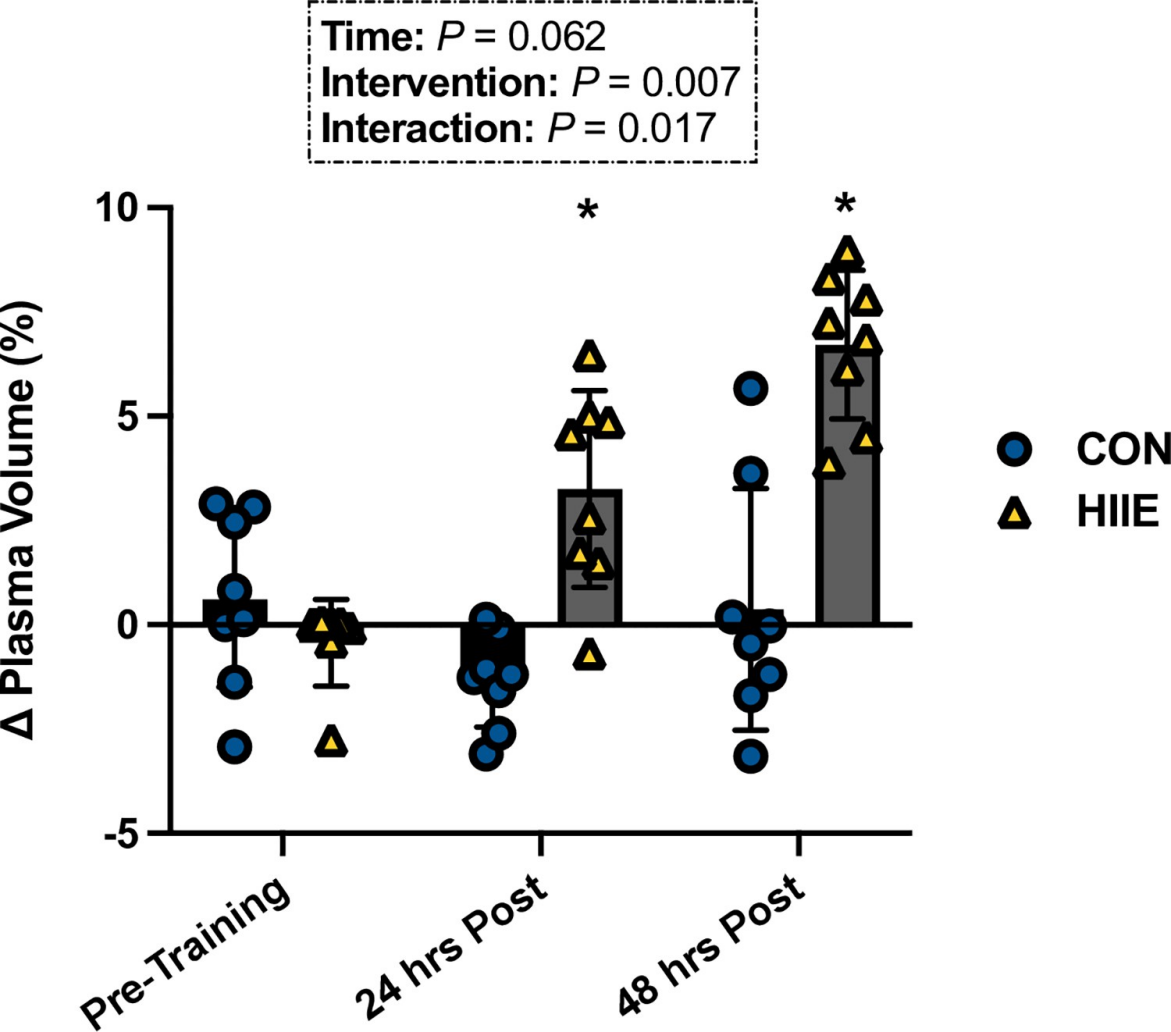
Changes in PV (n = 8) 24 and 48 hours following each intervention (HIIE vs. CON). Bars represent mean values for each intervention overlayed by individual values. * Denotes significant differences between interventions at each time point (*P* < 0.05). Data were analyzed using a two-factor (intervention x trial) repeated measures ANOVA. When a significant main effect or interaction was identified, we performed the Holm-Sidak post-hoc test to determine where differences existed. n = 8 for all comparisons.

Changes in PV throughout the study are shown in Fig 2. There was a significant interaction for PV (*P* = 0.017), which differed between HIIE and CON at 24 hours (*P* = 0.031; d = 0.49) and 48 hours (*P =* 0.006; d = 0.71). No differences for ΔPV were observed between HIIE and CON for pre-training (P = 0.128).

**Fig 3:**
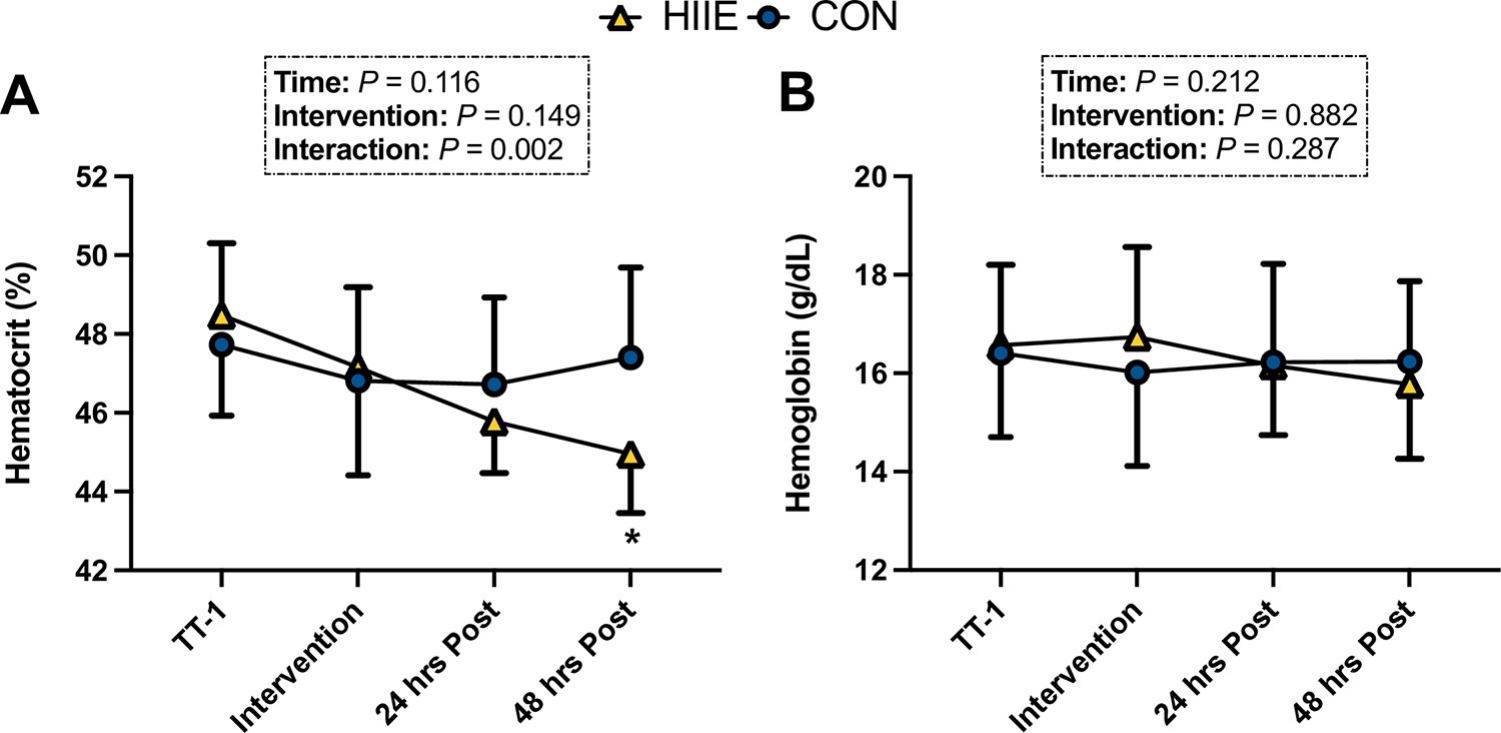
**A.** Hematocrit values (n = 8) for all timepoints in which blood sampling occurred. **B.** Hemoglobin values (n = 8) for all timepoints in which blood sampling occurred. Data are expressed as means ± SD.* Denotes significant differences between interventions at each time point (*P* < 0.05). Data were analyzed using a two-factor (intervention x trial) repeated measures ANOVA. When a significant main effect or interaction was identified, we performed the Holm-Sidak post-hoc test to determine where differences existed. n = 8 for all comparisons.

There was a significant interaction for Hct (*P* = 0.002), which differed between HIIE and CON at the 48 hours post timepoint (*P =* 0.030; d = 0.51; Fig 3A). There were no observed effects of time (*P* = 0.212) or intervention (*P* = 0.882) for Hb (Fig 3B).

### Hypoxic cycling time-trial

Averaged hemodynamic parameters for individual TTs are denoted on Table 2. There was a main effect of trial for HR (*P* = 0.008; d = 0.3), but no intervention (*P* = 0.491) or interaction (*P* = 0.844) effects were observed. There was an interaction effect for SV (*P* = 0.032; d = 0.4), which differed between HIIE TT-1 and TT-2 (*P* = 0.029, d = 0.4) and HIIE TT-2 and CON TT-2 (*P* = 0.007; d = 0.5). There was an intervention effect for $\dot{Q}$ (*P* = 0.023; d = 0.3), which differed between HIIE TT-1 and TT-2 (*P* = 0.047; d = 0.4) and HIIE TT-2 and CON TT-2 (*P* = 0.027; d = 0.7). RPE_final_, SpO_2,_ BL_Rest_, BL_peak_, and USG were not different between HIIE or CON and there were no differences between participant responses at TT-1 and TT-2 (all *P* > 0.05) (Table 2).

**Table 2:** Hemodynamic, perceptual, and physiological responses to 15 km hypoxic TT.

	TT-1		TT-2	
	HIIE	CON	HIIE	CON
HR (bpm)	165 ± 7.8	167 ± 9.6	**164 ± 6.6** *	165 ± 11.2
SV (mL)	125 ± 35	122 ± 36	**133 ± 34** †	122 ± 36
Q (L/min)	20.5 ± 5.0	20.2 ± 5.4	**21.9 ± 4.8** **	20.1 ± 5.2
RPE_final_	17.0 ± 1.7	17.5 ± 1.9	17.0 ± 1.0	16.6 ± 1.2
SpO_2_ (%)	87.3 ± 1.9	86.3 ± 2.4	86.6 ± 1.4	86.7 ± 1.9
BL_Rest_ (mmol/L)	2.0 ± 0.6	1.8 ± 0.4	2.1 ± 0.7	2.4 ± 0.7
BL_Peak_ (mmol/L)	13.8 ± 2.9	13.5 ± 2.6	14.5 ± 3.9	14.9 ± 2.8
USG	1.010 ± 0.006	1.007 ± 0.004	1.009 ± 0.006	1.008 ± 0.003

Averaged (n = 8) hemodynamic parameters for each hypoxic TT. Data are presented as mean ± SD. USG reflects pre-exercise measurement.*Trial effect (*P* < 0.05).

**Intervention effect (*P* < 0.05).

†Interaction (intervention x trial) (*P* < 0.05). Data were analyzed using a two-factor (intervention x trial) repeated measures ANOVA. When a significant main effect or interaction was identified, we performed the Holm-Sidak post-hoc test to determine where differences existed. n = 8 for all comparisons.

**Fig 4:**
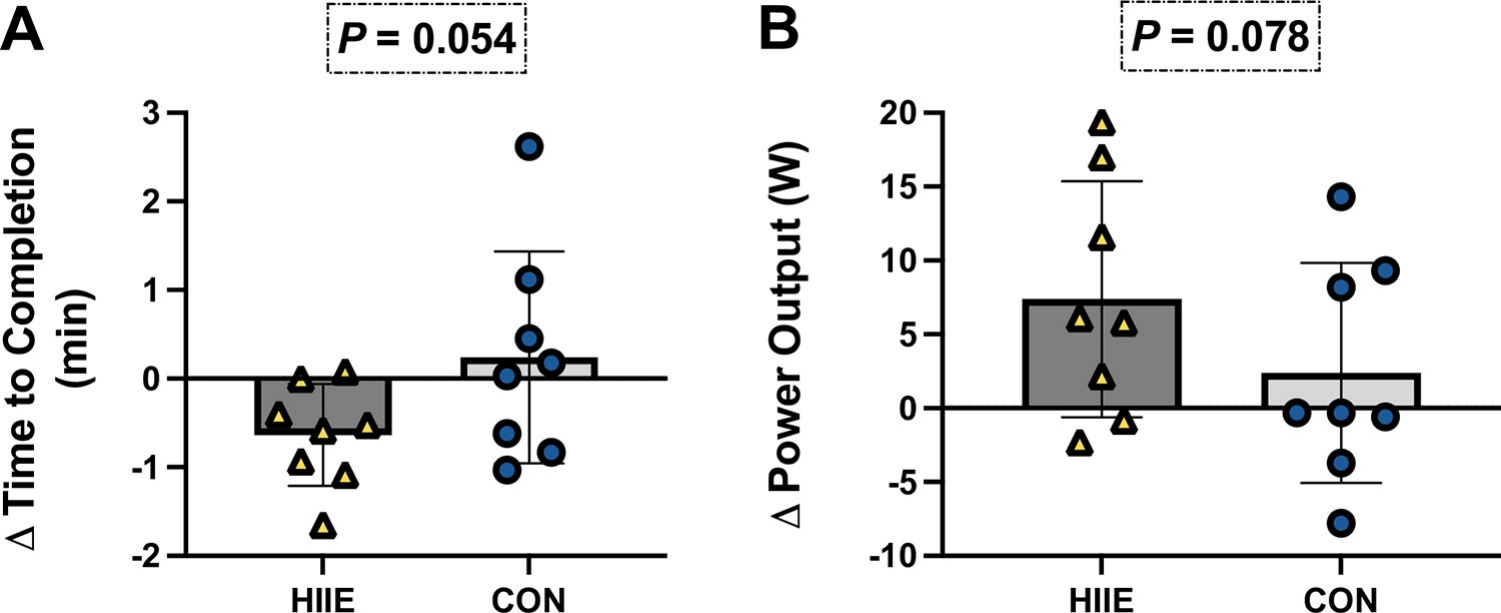
**A**. Changes in time to completion of 15-km TT between TT-1 and TT-2; **B**. Changes in average power output during 15-km TT between TT-1 and TT-2. Bars represent mean differences between TTs for each intervention overlayed by individual values. Data were analyzed using two-tailed Wilcoxon matched-pairs, signed rank tests. n = 8 for all comparisons.

Considering all subjects (n = 8), Wilcoxon matched-pairs signed rank test revealed no differences in change in time to completion of TT (−0.63 ± 0.57 min, *P* = 0.054; d = 0.4; Fig 4A), as well as change in averaged self-selected power output (7.37 ± 7.98 W, *P* = 0.078; d = 0.7) for TTs between interventions (Fig 4B).

## Discussion

Our main findings are that HIIE increased PV at 24 and 48 hours post exercise (Fig 2), as compared to CON, even though the interventions were matched for total workload. Elevated PV improved hemodynamic responses during TT hypoxic exercise, as evidenced by the significant increases in SV and $\dot{Q}$ without noticeable changes in HR (Table 2). Despite this, there were no differences in time to completion and power output during the TT (Fig 4). These data suggest that HIIE-induced PV expansion lowered cardiovascular strain, and may be an effective strategy to those seeking to mitigate decrements in exercise capacity in moderate hypoxia, without prior acclimation.

### HIIE and PV expansion

Exercise training leads to an increase of blood volume, which appears to be primarily accounted for by PV expansion [28]. Cross-sectional analysis of literature indicates PV can be increased within 24 hours following exercise and achieve ~10% increase from pre-exercise levels within 1 to 4 days [29]. The mechanism of hypervolemia associated with acute exercise-training is speculated to be congruently regulated by fluid retention (via renin-angiotensin-aldosterone system) [30], and increases in plasma albumin content [31], as these proteins account for 60–80% of the colloid osmotic pressure in the intravascular space [32].

Our investigation utilized a single HIIE bout, as we sought to isolate intensity-derived PV expansion exclusively developed from our independently matched interventions. To ensure that no changes in PV were due to exercise in hypoxia, PV expansion was measured between TT-1 and HIIE or CON and no differences were revealed (HIIE = -1.02 ± 1.94%, CON = 0.60 ± 2.09%; *P* = 0.128). Indeed, these data demonstrate that a significant increase in PV was observed 24 (4.05% ΔPV) and 48 (6.87% ΔPV) hours following the HIIE intervention only, further validating existing work documenting the rapid induction of hypervolemia with high-intensity exercise [19, 32, 33]. Accordingly, the degree of PV variation observed in our findings closely resembles those found in the literature surrounding blood volume changes, as Nagashima et al. induced a 6.4% increase in PV 22 hours following a similar upright exercise bout, whilst Gillen’s group reported a 10% expansion of PV following a similar treadmill high-intensity exercise bout [19, 32]. Furthermore, the absence of an environmental stressor in the current endeavor points to exercise intensity’s pivotal role as a signal for PV expansion, as previously suggested by others [31, 34]. Our findings further corroborate these outcomes, as demonstrated by the lack of ΔPV following the total workload matched CON intervention (-1.20% ΔPV 24 hours post CON; -0.20% ΔPV 48 hours post CON). As a consequence of the augmentation in PV following the HIIE intervention, we observed decreases in Hb and Hct concentrations, both at 24 (Hb = - 5.00%; Hct = -2.91%) and 48 (Hb = - 5.57%; Hct = -4.67%) hours post intervention.

### Hemodynamics

The observed PV expansion altered the cardiovascular response to TT performance in acute hypoxia. Improved SV and $\dot{Q}$ following HITT-induced hypervolemia are expected to be a product of increased left ventricular end-diastolic volume [35], speculated to arise due to enhanced central venous pressure [36]. There are data to support our findings of reduced cardiovascular strain (increased $\dot{Q}$ whilst maintaining exercising HR) following different dosages of known PV expanders (Dextrain or Pentaspan) [37, 38], aerobic training [39], and a single bout HIIE protocol resembling our study [40]. These improvements in hemodynamic variables were observed in different testing protocols at various exercising intensities, including at rest [40], during 90 minutes of moderate intensity exercise (62% of $\dot{V}O_{2\mathrm{peak}}$) [38], and at different time points throughout a 2-hour low intensity (46% of $\dot{V}O_{2\mathrm{peak}}$) cycling bout [37]. Our data are congruent with these studies, but we extend these findings into a hypoxic environment during TT performance. Interestingly, this increase in $\dot{Q}$ is not a consistent finding in literature, as two previous studies have found no effect of artificial PV expansion on $\dot{Q}$ and HR during submaximal exercise, despite observing transient increases in exercise SV [12, 41].

Alongside this reduction in cardiovascular strain, we observed decreases in Hb (24 hours post HIIE = -5.00%; 48 hours post HIIE = - 5.57%) and Hct (24 hours post HIIE = - 2.91%; 48 hours post HIIE = - 4.67%) concentrations (Fig 3). This is an important finding that likely corroborates previous reports suggesting that increases in SV and $\dot{Q}$, induced via acute PV expansion, are capable of offsetting reductions in Hb and Hct concentrations, such that muscle oxygen delivery is preserved during high intensity exercise [12, 17]. As such, this study highlights that intensity-mediated PV expansion is rapid, which may expedite performance changes when compared to traditional altitude acclimatization protocols that often require ~2 weeks of intermittent hypoxia exposure [42].

### TT performance

Various groups regularly travel to moderate altitude (2,000–3,000 m) for competition, recreation, or occupational tasks. The negative effects of hypoxia at this level of altitude are apparent, rapid, and well documented: a 7% reduction in aerobic performance [43], 12% loss in $\dot{V}O_{2\mathrm{peak}}$ [44], and an increase in submaximal HR [45]. Thus, there is a need to better understand countermeasures to exercise in acute hypoxia.

Various investigations have shown significant improvements in exercise performance attributed to an acute expansion of PV, making it a relevant adaptation for recreational endurance athletes to consider prior to competition [15, 16, 46, 47]. These data suggest that an enhancement in PV may be of benefit for those seeking incremental improvement of athletic performance at altitude. We observed only a trend in TT performance (time to completion: *P* = 0.054; d = 0.4; power output: *P* = 0.078; d = 0.7) despite robust increases in PV 24 and 48 hours post interval exercise. While the p values did not cross the *a priori* threshold for significance, the medium to large effect size suggest an improvement in cycling performance was realized. Though our findings differ from previously mentioned reports, our study was not sufficiently powered to directly investigate improvements in exercise performance. Further, data should be interpreted with caution since our participants were recreationally active and thus not accustomed to time trial performance. Our design incorporated a familiarization session prior to the first TT to remove the learning effect of pacing, but the inexperience of participants could have impacted our TT results.

## Perspectives

Given the wide variety of endurance sport competition offerings at moderate to high altitudes, questions regarding timing of arrival at altitude are continuously asked by professional and recreational athletes alike. Particularly, one arrival strategy commonly used by national soccer teams [48] termed “fly in, fly out”, proposes that arrival at altitude should be as close as logistically possible to the event start time, in order to minimize the acute reductions in PV associated with altitude exposure [49]. Previous investigations have observed ~5% reductions in PV within 14 hours of simulated 16% F_i_O_2_ [50], and > 10% reductions in PV within the first 24 hours at 4,000m of elevation [51, 52]. Reductions in PV are beneficial in increasing the relative concentration of Hb per liter of blood to preserve arterial oxygen content. However, this reduction concomitantly serves to elevate cardiovascular strain via reductions in SV, which can negatively impact exercise performance at altitude [53]. Nevertheless, while the reduction in PV is well-documented upon exposure to > 3,000 m, the minimal altitude at which these reductions take place remain unclear. Reports suggest that exposure to lower altitudes (defined as < 2,500 m) seem to have a minimal effect on PV [45, 54, 55]. In such cases, our findings suggest that an expansion of PV may be a beneficial strategy to partially mitigate the deleterious effects of hypoxia on the cardiovascular system, particularly when exercise will be initiated promptly upon ascent.

## Limitations

Few people are afforded the resources to properly acclimate to hypoxia. Given that we sought to evaluate the effectiveness of strategies employed at sea level in better preparing individuals for the enhanced metabolic demands of exercise at altitude, there are inherited limitations associated with this design. The results of this investigation are limited initially by the relatively small cohort (n = 8) and exclusive use of a male population. The choice to exclude females from the study was deliberate as it is speculated that the significant fluctuation in ovarian hormones throughout the menstrual cycle can impact blood volume following dynamic exercise [56]. These variations could confound re-creation of our results within a similarly trained female population. Also, the participants could not be blinded to the intervention, as the awareness of intervals was impossible to overcome. Thus, some participants may have expected to perform better after intervals compared to after control. Furthermore, the use of impedance cardiography to assess hemodynamic variables needs to be acknowledged. Previous work has highlighted potential limitations in the use of this technique [57], specifically regarding appropriate placement of electrodes and artifacts from neighboring organs. Despite these concerns, this device and its validity during rest and exercise testing have been previously published [58]. Additionally, placement of electrodes, calibration of device, as well as extraction of the collected data were performed in accordance with the manufacturer’s instructions. Finally, our participants were recreationally active and thus their PV response to HIIE may differ compared to highly trained athletes, who have already expanded their plasma through habitual aerobic training.

## Conclusion

A single cycling bout of HIIE is an effective strategy to induce hypervolemia within 24- and 48-hours following exercise. In the present study, this expansion in PV served to increase stroke volume and cardiac output, while lowering HR. The utilization of high intensity intervals prior to athletic endeavors may be an alluring strategy to increase cardiac output during exercise in hypoxia.

## Abbreviations

BL: Blood Lactate
CON: Control
Hb: Hemoglobin
Hct: Hematocrit
HIIE: High Intensity Interval Exercise
HR: Heart Rate
PV: Plasma Volume
$\dot{Q}$: Cardiac Output
RPE: Rating of Perceived Exertion
SpO_2_: Arterial Oxygen Saturation
SV: Stroke Volume
TT: Time Trial
USG: Urine Specific Gravity
$\dot{V}O_{2\mathrm{peak}}$: Maximal Oxygen Uptake
